# Supervising international students in clinical placements: perceptions of experiences and factors influencing competency development

**DOI:** 10.1186/s12909-016-0702-5

**Published:** 2016-07-16

**Authors:** Stacie Attrill, Michelle Lincoln, Sue McAllister

**Affiliations:** The University of Sydney, Adelaide, Australia; Flinders University, Adelaide, Australia; Speech Pathology and Audiology, Flinders University, GPO Box 2100, Adelaide, SA 5000 Australia

**Keywords:** Clinical placement, Competency, Cultural diversity, Focus groups, International students, Internationalization, Supervision

## Abstract

**Background:**

Health professional education programs attract students from around the world and clinical supervisors frequently report that international students find learning in clinical placement contexts particularly challenging. In existing literature clinical supervisors, who support international students on placement have identified concerns about their communication and interactions within clinical environments.

However, clinical supervisors’ perspectives about their experiences with international students on placement and the strategies they utilise to facilitate international student learning have not been described. As a result we have little insight into the nature of these concerns and what clinical supervisors do to support international students’ competency development.

**Methods:**

Five focus group interviews were conducted with twenty Speech-Language Pathology clinical supervisors, recruited from 2 Australian universities. Interview data were analysed thematically. Themes identified were interpreted using cognitive load and sociocultural learning theories to enhance understanding of the findings.

**Results:**

Four themes were identified: ‘Complex teaching and learning relationships’, ‘Conceptions of students as learners’; Student communication skills for professional practice’, and ‘Positive mutual learning relationships’.

**Conclusions:**

Findings indicated that clinical supervisors felt positive about supporting international students in clinical placements and experienced mutual learning benefits. However, they also identified factors inherent to international students and the placement environment that added to workload, and made facilitating student learning complex. Clinical supervisors described strategies they used to support international students’ cultural adjustment and learning, but communication skills were reported to be difficult to facilitate within the constraints of placements. Future research should address the urgent need to develop and test strategies for improving international students’ learning in clinical settings.

**Electronic supplementary material:**

The online version of this article (doi:10.1186/s12909-016-0702-5) contains supplementary material, which is available to authorized users.

## Background

Health professional education programs attract students from different countries and therefore many cohorts will include international students (IS) who are not residents of their country of study. Clinical placements are central to health professional education programs. They support students to develop professional competencies and attributes, and embed them within the sociocultural practices of their profession and the broader healthcare team [1, 2]. Clinical supervisors, from here referred to as ‘supervisors’, manage and supervise students’ learning during clinical placements, working closely with them to support, shape and assess their competency development [3]. IS may find learning in clinical placements challenging [4–6]. This may be influenced by distinctive experiences of learning and education in their home countries [7–9], their understanding of the health and community services in which clinical placements are situated [6, 10], and communication and cultural adjustments required for successful interactions with the range of people encountered in clinical settings [6, 10, 11]. Supervisors therefore are assisting IS to manage the learning demands associated with these factors [11]. Studies that have explored IS’ learning in clinical placements have focused on perceived challenges related to IS’ communication skills or culture, or on the students’ experience of placement. However, supervisors’ perspectives on their experiences with IS on placement, and the strategies they utilize in placement to support IS’ learning have not been described. As a result we have little insight into the nature of supervisors’ concerns and how they attempt to address them.

Literature about IS’ learning in health professional placements has been situated within Western, English speaking contexts. In allied health and nursing studies, supervisors perceived culturally and linguistically diverse (CALD) students, including IS, as challenging, requiring greater support and supervision time on placement than their peers [6, 12, 13]. Whilst non-native English speaking IS have been reported as likely to fail a placement [10], studies have not clearly differentiated outcomes for IS from CALD students, a group which includes domestic students [6, 12, 13]. However, IS may have different learning needs to CALD domestic students [9, 11]. Remedial suggestions such as English language courses may not adequately address broader supervisor concerns about IS’ clinical interactions and understanding of the Western health context [4, 6]. Supervisors may therefore need to identify and implement unique support strategies for IS, adding to their perceptions of increased workload.

Supervisors and IS have identified cultural differences as contributing to IS’ difficulties in clinical placements [10, 11]. IS themselves have identified that their different cultural background creates challenges for learning in placements [11]. Supervisors’ perceptions may relate to their understanding of cultural issues and how these may influence supervisory relationships. Successful supervisory behaviours for culturally diverse students include promoting cultural identity among students, being aware of own culture, being positively responsive to cultural issues and creating a safe environment for discussion about these [14, 15]. To competently enact these behaviours within clinical settings, supervisors may need well developed cultural knowledge and skills and understand how to apply these to support students’ learning. This may contribute to supervisor perceptions that IS are challenging to support. However, previous research has not explored the connections between supervisors’ perceptions of challenge and their experiences supporting IS on placement.

In clinical placements, the complex and changing needs of clients have primacy over student learning [1, 4]. Furthermore, supervisors manage their clients’ needs while observing, making judgements about and facilitating student learning [16, 17]. Processing information relating to their students and clients creates high cognitive load for supervisors [17, 18]. For example, GP consultation time was found to be the same when students were present, but the GP’s perceived consultations as longer. The authors hypothesized that GPs engaged in complex activities when meeting both patient and student’s needs contributing to their perceptions of time spent [19]. Reasoning and problem solving about student learning may also be affected by contextual factors related to the supervisor, setting and student [18, 20]. An investigation of the influence of contextual factors on medical internist’s clinical reasoning found that their cognitive load increased for patients with English as a second language (ESL) [20]. Variables such as cultural background or English proficiency are contextual factors related to IS that may also add complexity to supervision. This may add learning and attention demands for supervisors, potentially influencing their perceptions of challenge and time.

The sociocultural context may also mediate supervision practices, influencing supervisor teaching strategies. Supervisors use their knowledge and professional identity to support students’ learning. This identity is formed through the supervisor’s membership of their professional community [2, 21] and will include cultural constructs and values about the nature of clinical practice. These guide how supervisors facilitate students’ development of competency [8, 22]. Their teaching preferences are also shaped by personal and professional learning experiences, and the setting in which the placement occurs [23]. It may be more challenging for supervisors to teach IS on placement if the students’ preferences, experiences and values about learning differ from their own [21].

Universities have incorporated systematic processes to internationalise their curriculum to facilitate IS’ learning and provide enriching intercultural learning opportunities for all students. Internationalisation aims to integrate international, intercultural and global dimensions into teaching and learning, and is underpinned by principles of equity and inclusion to benefit all learners. Strategies developed to assist IS include removing perceived cultural biases, using inclusive language and allowing additional time for examinations [7, 8]. However, it may be challenging for supervisors to employ internationalisation strategies within the client focused settings in which placements are situated [1, 4], and this has not been investigated in previous research.

### Aims of the study

To date, studies have addressed IS’ skills or experiences [11, 13] or their perspectives about placement supervision [15]. As supervisors in previous studies have perceived CALD students as challenging to teach and requiring more supervision time than their peers [6, 12, 13] it is possible that IS present greater challenges requiring advanced skills to effectively supervise them [14]. This may reinforce supervisors’ perceptions of increased workload, resulting in reluctance to supervise these students. Effective strategies for supervisors to utilize when teaching IS in placement may improve the placement experience for all stakeholders.

This study aimed to contribute in-depth qualitative description of supervisors’ perceptions of their experiences facilitating IS placements and the teaching strategies they report using within placement settings.

This study addressed the following research questions:

1. What are the perceptions of supervisors about their experiences with IS on placement?
2. What strategies do supervisors report using to assist the competency development of IS?

## Methods

### Context of the study

This focus group study was conducted with supervisors of Speech-Language Pathology (SLP) students in Australia enrolled in either a 4–year undergraduate or 2- year Masters professional preparation program. Students complete a number of clinical placements over 1–2 years in the latter period of their program. They are placed within healthcare, education and community settings and participate in direct service provision in collaboration with their supervisor, taking more responsibility for client care as their competency develops.

The supervisor facilitates the student’s developing professional skills through observation, feedback and discussion. Continuous formative assessment of competency development occurs over the placement, guided by a standardised competency assessment tool on which the final summative assessment is recorded [24]. Supervision of 1 or 2 students for the duration of a placement is most common in Western SLP programs [3]. The majority of supervisors (>90 %) access non-mandatory supervision training on principles of workplace learning and assessment, including preparing for placements, goal setting and feedback [25].

For the purpose of this study, IS were defined as ‘overseas residents enrolled in an Australian onshore higher education program’ [5]. Recent data suggests that approximately 8 % of students in Australian SLP programs are IS, and this proportion is anticipated to increase in the coming decade [26, 27]. These students are predominantly from South East and Southern Asia, North America and the United Kingdom. Supervisors in this study had supervised IS with a minimum International English Language Testing System (IELTS™) score of 7.0, which is defined as a good English user [26, 28].

### Participants

Supervisors were purposively sampled from SLP student placement records from 2 universities (A and B), in separate Australian states. They were emailed information about the study if they had supervised at least 1 IS on placement during the previous 2 year period and participants provided written consent. Participants were all females from an Anglo background, reflective of Australian SLPs who are 97.5 % female and 88 % Anglo-Australian [29]. They had IS supervision experience ranging from 2 to more than 10 years of experience, encouraging diverse discussion to inform thematic development [30]. Table 1 shows the number of supervisors who were approached, those who consented and those who were available and then participated in a focus group.

**Table 1:** Supervisors consented, interview participation and focus groups conducted

	University A	University B	Total
No. focus groups conducted	3	2^a^	5
Supervisors invited to participate	41	29	70
Supervisors consented	19	10	29
Supervisors interviewed	13	7	20

^a^One focus group was conducted via teleconference

### Procedures

#### Interviews

A social constructivist approach was taken using focus group interviews to generate and collect data. These enabled supervisors to compare and contrast their experiences of supervising IS together, and construct their understanding of their common experiences, perceptions and strategies [31]. Interactions between participants provided rich data not accessible via individual interviews, which was useful to explore supervisors’ insights [32].

Five focus groups of approximately sixty minutes were conducted in 2011–2012, comprising 3 to 5 participants each. Table 1 shows the number and location of the focus groups. The first author was involved with assessing students with participants from University A, but no such relationship existed with participants from University B. Therefore, the third author conducted focus group interviews at University A and the first author conducted focus groups at University B. Both researchers had prior experience conducting qualitative interviews.

The interview guide was informed by themes in the literature and the research questions (see Additional file 1). Four topics were discussed: Experiences with IS on placement; perceptions of IS competency development; assessment of IS; and strategies to support IS learning. Interview questions progressed from general to more specific, and interviews were audio recorded and transcribed verbatim. Participants were sent the interview transcripts for review, but no feedback was received. Data was managed using NVivo 10.2 software. The first author listened to each interview and wrote memos about the conversation, topics and interactions within and between groups [33].

### Analysis

Krueger and Casey’s (2009) classic analysis framework was combined with Braun and Clarke’s (2006) procedures for thematic analysis enabling rich description of the dataset. Inductive inquiry was undertaken, so open coding was used to describe the transcribed data and occurred concurrently with subsequent interviews. Coded data were sorted into interview topics, and then categorised as demonstrated in Table 2 [30, 33, 34]. Information about group interaction was included in codes and memos [32].

**Table 2:** Open coding and categorisation of transcription data according to interview topic

Interview topic	Raw transcript data	Open code generated	Category
*Placement experience*	*“I think they’ve taught me a lot about the way I communicate”*	IS teach me about the way I communicate	IS inform my teaching and practice
	*“Trying to unpack what it was that I was doing differently, or what it was that I was doing, which was quite interesting for me to think about as well”*	Interesting for me to think about unpacking what I was doing	
*Competency development*	*“Communication was difficult because of their English as a second language issues. So I guess it was specific to them being international”*	Communication issues related to ESL were specific to IS	English language competency
	*“For me it’s about the quality of the English spoken”*	Challenges re quality of the English language spoken	

Themes were identified by examining and grouping categories, guided by the classic analysis framework [34]. Constant comparison of the categories ensured that the themes were internally consistent and grounded in the data [30, 33]. The authors discussed the themes and reached consensus. Interpretation of the themes was guided by cognitive load and sociocultural learning theories to develop an understanding of supervisors’ perceptions of IS in placements [2, 18, 21]. The researchers were female SLP university educators with substantial supervision experience, including with IS. These researcher characteristics were known to the participants. Whilst themes were identified inductively, the researchers’ background inevitably influenced data analysis.

### Rigour

An emergent-systematic focus group design was employed [30], where University B interviews were conducted to verify categories from University A. The same categories existed within and across datasets, and data saturation was achieved. The 3 authors independently categorised open codes from the interview topic ‘Experience with IS on placement’ [30]. Categories were discussed, minor discrepancies resolved, and consensus achieved. The first author analysed the remaining dataset which is included as Additional file 2.

Participants from University A focus groups were invited to join a subsequent focus group to verify the themes. Ten participants consented and 6 were available for the interview. Participants agreed with the themes, and explored these with examples from their own experiences. These examples existed in the data, and no new ideas or concepts were identified.

## Results

Participants narrated many examples from their experiences and a range of perspectives were gathered. Consensus was high across the groups, including for negative perceptions. Four themes were identified from data analysis, which are shown with their major categories in Table 3. Overall, themes described the supervisors’ perceptions of managing and facilitating the IS’ competency development, including their perceptions of how IS enact their learning and communication skills for professional practice, and beneficial features of their supervision experience. The themes are reported in the findings in order of their prominence in the data.

**Table 3:** The relationship between themes and their categories

Theme	Category
Complex teaching and learning relationships	Supervisor pressure and responsibility re IS performance
	Knowing how to help students
	IS problems on placement are challenging and complex
	Adjusting to working in different systems, with people from different cultures
	IS manage added complexities and pressures
Conceptions of international students as learners	Attitudes, behaviours, values and expectations as learners
	Students are individuals
	General professional skills
Student communication skills for professional practice	English language competency
	Professional Writing
	Social and interpersonal skills
	Communication skills impact multiple competencies
Positive mutual learning relationships	IS inform my teaching and practice
	Cultural exchange, learning and enrichment
	Positive experience
	IS working positively with clients from CALD backgrounds
	Clients assist IS learning

### Theme 1: Complex teaching and learning relationships

Participants identified that their teaching and learning relationships with IS were complex, adding workload and time. They often perceived pressure and responsibility associated with ensuring positive learning experiences for IS, maintaining quality services for clients working with IS, and adherence to the professional standards required for students to progress. Many participants also described stress and discomfort about their role as competency assessors for IS, expressing feelings of guilt when students failed placements.

“One of my biggest challenges …is the intense guilt I feel when I do fail them. When they’ve come from another country, paid more money, had to pay rent, are here by themself” (Interview 4, Participant 2).

Participants recognised that enacting services for clients was complex for IS compared with their domestic peers. IS often worked with clients from unfamiliar cultural backgrounds, within the foreign operations of their placement setting. The requirement to deliver services in English was also noted to add complexity for non-native English speaking IS. Participants suggested that there were different cultural, social and financial implications for IS of not succeeding in their studies compared with domestic students. These aspects were emotionally engaging for participants, which added complexity to their task of making objective judgments about student competency.

“If they’re struggling with English as a second language, different cultural norms about how you interact with other people. Different cultural expectations about what they achieve academically, and expect of themselves. They’re potentially away from their secure base at home to help them regulate all of that. I think those all add many layers of complexity to the task of learning” (Interview 1, participant 3).

Participants reported that managing IS’ learning needs was difficult and impractical in clinical settings and added further complexity and workload. They discussed managing multiple additional demands such as providing client services, participating as a clinical team member and supervising other students. Supervisors identified strategies they used more frequently with IS to manage their competency development in the complex placement learning environment. These included requiring written session plans, step-wise modelling and elaboration of techniques to use with clients, encouraging repetition and practice, and providing specific, timely feedback in verbal and written modalities.

“I tend to give quite a bit of written feedback, but I think that I found with the international students, if I wanted something or if I had specific feedback, to write it down. I thought, kind of helped as well, so that they had it there, they could look over it again later. If it is a second language it is harder to do, when you’re processing and stuff at the same time.” (Interview 1, participant 5)

A student’s cultural background also added complexity to the learning relationship. Many participants considered culture to be as important an influence on the IS’ competency development as communication skills. Participants reported using strategies to scaffold IS’ adjustment for the culture of their clients and the workplace to enable IS to deliver services more effectively. Strategies included time spent with IS discussing their background and prior learning experiences, identifying IS’ cultural behaviours, and teaching IS about the clients’ cultural background. However, participants enacted these strategies for IS’ cultural adjustment in addition to usual strategies targeting students’ competency development. This added complexity to the supervisors’ task of facilitating IS’ learning.

“the cultural, it’s really important that we actually unpack that and spend time… more time with the international student because you anticipate bigger differences” (Interview 1, participant 3).

Most participants commented about feeling ill-prepared to support students whose learning was complicated by cultural or communication differences. University based learning supports provided for IS were sometimes perceived as helpful, but participants often reported IS were *“much more work”* (Interview 4, participant 2).

“You sort of go okay… I’ll put this in place, I’ll do this, I’ll change my language…. Nothing changes. So you almost feel like a failure, don’t you? What else can I do?” (Interview 4, participant 1)

### Theme 2: Conceptions of students as learners

Supervisors perceived that IS’ learning behaviours affected students’ competency development, and this influenced their perceptions of IS as learners. Many participants also described the impact of power, respect and their status as expert on their relationships with students from non-Western cultures.

“the compliance has been a kind of cultural respect and an unwillingness to actually argue a point or to explore a point further, which I feel I am obliged to do because I know that it [is] more beneficial to the student to actually think deeper” (Interview 5, participant 2)

Participants often considered successful students to be self-directed in their learning, confident to initiate interactions within clinical environments and able to work well with others and in groups. They commented that cultural barriers often inhibited IS’ ability to demonstrate these behaviours on placement.

“That sense of coming forward and asking a question or seeking support or clarification. We value that in terms of the competency of, (eg) ‘initiates learning, attitude to learning’. For somebody who’s culturally, that’s not common practice or that’s perhaps seen as rude or as showing incompetency, I’m actually judging them and saying ‘you demonstrate a poor attitude to learning’. Because you don’t come to me, you don’t ask questions, you don’t present a hypothesis, you actually just want me to direct you.” (Interview 4, participant 3).

Participants placed importance on facilitating the learning of all students as unique individuals. However, a tension existed as they held stereotypical belief’s about IS’ culturally influenced learning behaviours.

“There’s a bit of a danger in lumping [IS] all together, but at the same time, it’s important to have those cultural, not stereotypes, cultural issues in your mind” (Interview 1; participant 4)

Many participants described raising IS’ awareness of, and shaping expected learning behaviours for placement to enable IS to participate successfully in the learning activities of the placement. Target behaviours included asking questions, contributing to group processes and initiating discussions with supervisors about learning. Participants often discussed their expectations about these behaviours with IS and identified opportunities for IS to enact these.

“So that’s required a lot of talking and… ‘You know this is my expectation that you will ask questions, that you will contribute and that it may be something that you’re not comfortable or not used to doing’… Which is a really important part of a clinical process, is that participation in group conversations and unpacking what’s gone on, commenting, observing, questioning.”

### Theme 3: Student communication skills for professional practice

The development of communication skills appropriate for Western clinical contexts was considered a complex task. Participants considered this crucial for successful interactions with clients, team members, peers, and to support their own learning relationship with the IS. They identified communication skills related to IS’ proficiency with English, including features such as accent, grammar and vocabulary.

“It seems that there’s a theme that if students have competent English then that makes life easier for them and for you and for clients, and with the competencies” (Interview 1, participant 3).

English skills were widely considered to distinguish IS with competency difficulties from those without. This was illustrated particularly by comparing native and non-native English speaking IS.

“They’ve been from Canadian backgrounds. So they can speak English as a first language. The only real differences have been sounds and word choices” (Interview 5 Participant 1).

Participants reported teaching IS strategies for interpersonal client interactions, for example, interpreting clients’ use of humour, or helping to identify conversation topics. Many participants identified that assisting IS to develop professional communication skills, such as understanding clinical interactions, managing the impact of their accent and professional writing skills during a placement was challenging.

“I think the other thing from my perspective is knowing how to help them.” (Interview 4, participant 3). “Exactly, I think that’s the biggest thing” (participant 1).

Long term strategies were frequently suggested as necessary to support IS’ professional communication skills. However, implementing these were beyond the temporal and practical boundaries of the placement. Participants had recommended strategies to IS such as viewing English television programs, interacting with domestic students and accessing university English language services. However, participants often expressed dissatisfaction with these strategies, reporting that their attempts to support IS to improve their communication competency were limited or futile.

“They can’t change it in 6 months. They probably need 3 or 4 years of going away and practicing… before they’re competent enough to come back and give it a go in a high level language situation with a… client. They don’t have that time. That’s the problem” (Interview 4, participant 2).

### Theme 4: Positive mutual learning relationships

IS frequently assisted supervisors, clients and peers to develop greater awareness of culture and their own attitudes to students from diverse backgrounds. Many participants acknowledged the mutuality of the learning relationship with IS, and viewed this positively. Some participants used this learning to further their cultural understanding, applying this in clinical practice and with other students.

“The international students make me think a lot more about my attitude as a white Anglo Australian” (Interview 2, participant 1)

Participants considered IS’ understanding of culture and language an asset to their practice, valuing the new ideas and perspectives they brought to client work. Some participants also reflected about positive adjustments to their teaching and client practices made in response to their supervision experiences with IS and the strategies they had enacted.

“Where there [are] complexities of language and culture, for me it’s helped really clarify and it made me explain, shed more light” (Interview 1, participant 4)

Mutual benefit was also evident for clients. Many participants narrated stories about positive outcomes and teaching opportunities for clients working with IS. These occasions for mutual learning and teaching fostered confidence and goal attainment for students and clients.

“I’ve seen adults and children try to help out… That’s a lovely interaction that’s reassuring to the international students and probably confidence boosting to the patient or child” (Interview 2, participant 5).

## Summary of the Findings

Together, these findings described supervisors’ perceptions of the complex nature of supporting IS on placement. Supervisors perceived that IS added to their workload and time, but were also aware that for IS, the language, learning and cultural context in placements is complex.

Supervisors acknowledged that their judgments about IS’ skills often related to communication, culture and students’ prior learning experiences, complicating their teaching. They identified strategies to facilitate IS’ adjustments for culture and learning in the placement, but strategies to support communication competencies were perceived as less effective. However, most supervisors were positive about their experiences with IS. They acknowledged a benefit to their own practice and cultural skills as well as how the intercultural skills that IS bring to their work benefits clients and other students. Although these themes have been described separately, there were relationships between themes and the elements underpinning them. The combination of these elements were complex for supervisors, who were managing interrelated challenges incorporating culture, communication and learning with other work based demands. For example, when asked about factors influencing competency development, participant 3 from interview 3 replied:

“I don’t know if it was partly [an] English language or a cultural thing that we just weren’t able to get there, cos she was trying, she would do what she thought I’d asked and it just wasn’t… and I was having a lot of trouble.”

## Discussion

This study used focus groups to explore SLP supervisors’ experiences of facilitating IS competency development in clinical placements and strategies they utilised to assist IS’ learning. Four themes were identified: ‘Complex teaching and learning relationships’, ‘Conceptions of students as learners’; Student communication skills for professional practice’, and ‘Positive mutual learning relationships’.

The challenges captured in the first theme support previous research about supervisors’ concerns regarding CALD students on placement, including English proficiency, adjustment to educational expectations, social stress, and understanding foreign health settings [4, 6, 9]. Supervisors in this study were concerned about IS’ English proficiency. However issues of cultural adjustment and understanding of the service context were also commonly described including when IS had a western cultural background or were native English speakers. Participants identified that issues influencing IS competency development in placements were complex to manage and required extra time within their busy clinical contexts. Issues of complexity, time, stress and workload burden for supervisors when working with CALD students in placements are well represented in literature [6, 12].

However, supervisors’ observations of greater time to support IS may be perception rather than reality. Walters and colleagues suggested that their GP participants may have required different processing skills to meet the more complex combined needs of patients and students, compared with those used in solo consulting [19]. Durning and colleagues’ identified increased cognitive load for decisions regarding patients with added context, including ESL [20]. IS’ English proficiency and cultural adjustment adds contextual variables for supervisors already managing high cognitive load [17]. A link may exist between cognitive load and supervisors’ perceptions of time and complexity when working with IS. Further research is needed to confirm this relationship and whether strategies to support supervisors to manage this load enhance placement experiences for supervisors and IS. Strategies may be particularly helpful when student variables, such as English proficiency and cultural or learning differences add to the complexity of supervision.

The second and third themes capture supervisor perceptions that IS’ cultural behaviours and communication skills affect supervision relationships and interactions, and those with clients, peers and other staff members. Supervisors observed the influence of their power and expert status on the degree to which IS directed their own learning. Previous studies about IS have found their relationships on placement to be influenced by their cultural background and expectations of the student-teacher relationship [14, 15]. The current study also reinforces that behaviours related to IS’ culture and learning influence how supervisors interpret their learning. However, supervisors also noted variability in IS’ presentation and skills noting that it is not inevitable that they experience difficulty developing their competency. This may reflect the IS’ acculturation or learner preferences [35], or sociocultural factors including gender, family, and prior educational experiences [15, 22]. Supervisors may therefore need to generate individualised learning strategies for IS, rather than applying standard strategies for students from comparable backgrounds. This may be complex for supervisors to enact in placement settings.

Participants perceived positive learning behaviours for IS on placement as including demonstrating self-directed learning behaviours, asking questions and working well with others. Some participants perceived that for many IS, sociocultural conventions inhibited these behaviours from developing. Western educators may utilise a deficit model to understand IS learning, critically comparing them with domestic peers [8, 35]. This may reflect a supervisor’s own culturally bound understanding of learning as an interactive, social process, where they perceive their role as assisting students to construct knowledge and skills [23]. It is likely then, that complexity is increased for supervisors when IS have different learning expectations and behaviours to their own [8, 10].

Supervisors guide students to develop competency based on professional practices grounded within the supervisor’s own culture and values [2, 21]. IS may enact their professional skills with clients in ways typical of health professional practice in their own practice community. Supervisors accustomed to learners who behave according to their sociocultural norms and traditions may find facilitating IS' learning challenging [21, 22], adding complexity when student supervision tasks are inherently complex. Supervisors may need new understanding to accommodate the behaviours of students less reflective of the dominant culture. Development of theory about how supervisors understand learning and how this might differ for students from diverse backgrounds will assist to frame and test effective support strategies.

Participants identified English proficiency as characterising IS experiencing difficulties with competency development on placement and this has previously been noted as a factor in placement success for CALD students [12, 13]. In this study, participants identified similar areas of concern with IS’ language features including accent, vocabulary, and written English. They also reported supporting IS with conversation, non-verbal skills and culturally embedded aspects of communication, such as interpersonal interactions and humour. Communication is important in the provision of client care [4], so its prominence in this study is not surprising. However, supervisors in this study identified more communication features than in previous studies, and perceived that cultural factors influence how communication is enacted in clinical settings. Future studies could usefully examine interactions between IS’ cultural and communication skills to assist to identify targeted strategies for learning on placement.

In the final theme, supervisors identified positive mutual benefits of working with IS for themselves, clients and other students through opportunities for intercultural learning, and developing their own cultural awareness and attitudes. Whilst similar personal and professional benefits for supervisors working with IS have been identified [14], these have not been prominent in the clinical education literature, which has concentrated more often on IS deficits than their positive contributions. This study suggests that IS do contribute positively in the clinical placement context.

### Strategies to support international students

Recommendations for supervisors supporting IS on placement have been indirectly inferred from student perspectives or expert opinion [14, 15] or addressed CALD students rather than IS [6, 12]. Suggestions have included training and preparation for students and supervisors, targeting students’ English skills or modifying workload, but have rarely reported direct strategies utilized by supervisors in situ on placement. Participants in this study identified several strategies to support IS’ adjustment to the cultural and learning milieu of the placement, competency development and understanding of the operations of the setting. Strategies that facilitate IS’ adjustment for the placement may encourage trusting supervisory relationships [14, 15]. These included exploring the IS’ cultural background, learning preferences and behaviours, and identifying cultural features of communication. Enacting these strategies enabled supervisors and IS to collaborate about goals to facilitate adjustment and competency development. Supervisors also reported using teaching strategies such as modelling and elaboration of techniques for clients and use of written modalities for session planning and feedback more often with IS. This structure and direct guidance may reduce elements for IS to process, assisting them to manage cognitive load related to their adjustments and competency development [11, 17]. The strategies utilized by supervisors to facilitate and scaffold IS learning in placement are similar to strategies applied to internationalise the curriculum in university-based learning contexts. The mutual and intercultural learning benefits supervisors reported for themselves, clients and other students similarly resonates with intended internationalisation outcomes described in the literature, demonstrating they can be applied to placement settings [7, 8]. Many participants also suggested the need for training to develop their understanding of IS and assist them to identify effective support strategies. Training that acknowledges IS as assets to practice, reinforces their unique, rather than stereotypical presentations and shares supervision strategies may assist to reduce their perceptions of load and complexity.

In contrast to strategies for learning and cultural adjustment, participants described difficulties providing meaningful English and communication supports for IS within their complex clinical settings, and in the temporal constraints of placements. Participants noted that university supports are important to assist students to develop English proficiency for professional communication, given the longitudinal nature of language acquisition. However, this may be different to English supports provided by universities for academic success [35]. Efficacious strategies to support communication skills development for IS in placements have not been identified in the literature [4]. Future research should ensure any strategies identified are appropriate to implement within clinical settings.

### Limitations

This study was conducted with Western, English speaking SLP supervisors working with IS in Australian clinical placement settings. The findings may not generalise to other cultural contexts or health professions. Participants were city and rural supervisors from 2 university courses in separate Australian states who varied in the number of IS they had supervised. However, data about the amount and background characteristics of IS they had supervised was not collected, reducing the study’s replicability. All participants were female, but evidence suggests that men and women approach student supervision differently [23]. Findings may have varied if males were represented. In view of these limitations, the findings should be interpreted cautiously. However, the themes relate to a range of previously published research [12, 13, 15].

## Conclusion

This study explored supervisors’ perceptions about their experiences with facilitating IS learning on placement, and the strategies they use to facilitate IS competency development. From the findings, it can be concluded that supervisors felt positive about supporting IS and experienced mutual learning benefits. However, they also perceived factors inherent to IS and the placement environment that made facilitating IS' learning complex. Supervisors identified strategies they used to facilitate cultural adjustment and learning for IS, but noted that communication skills related to English language were difficult to support in placements. The findings suggest that differences in communication, culture and learning behaviours contribute to supervisor perceptions of complexity, and this may relate to their cognitive load and expectations about student learning. Future research to investigate how supervisors understand learners from diverse backgrounds would be useful. This will inform the development of effective strategies to support student learning both at university and on placement.

## Abbreviations

CALD, Culturally and linguistically diverse; ESL, English as a second language; IELTS, International English Language Testing System; IS, International students; SLP, Speech-Language Pathology

## Additional files

Additional file 1: Supervisor interview guide. (DOC 45 kb) Additional file 2: Thematic analysis dataset. (XLSX 130 kb)
