# Supplementary material for: Supervising international students in clinical placements: perceptions of experiences and factors influencing competency development
Source: BMC Med Educ. 2016 Jul 16;16:180. doi: 10.1186/s12909-016-0702-5 (PMC4947354; doi:10.1186/s12909-016-0702-5)
Supplement: Additional file 1: — Supervisor interview guide. (DOC 45 kb) [file 12909_2016_702_MOESM1_ESM.doc]

**Appendix A**

**Clinical Supervisor Focus Group questions**

Primary:

1. Tell me about your experiences working with international students on placement.
   1. What factors have influenced these experiences?

Secondary:

1. How do you feel about working with international students compared with local students?
   1. What factors influence these feelings?
2. Tell me about experiences that have caused you to feel positive about International students on placement with you?
   1. Why?
3. Tell me about experiences that have caused you to feel negative about International students on placement with you?
   1. Have you had particular challenges with international students on placement?
      1. What have been the biggest challenges?
4. Do you feel that students’ English language skills have influenced their experiences on placement?
   1. What has been your experience of this?
   2. How do you feel about this?
5. Do you feel that cultural factors have influenced students’ experiences on placement?
   1. What has been your experience of this?
   2. How do you feel about this?

Primary:

1. Tell me about your experiences with competency development for International students on placement.
   1. What have been the most important factors that have influenced competency development for International students on placement?
   2. Why?

Secondary:

1. Do you feel there are COMPASS® competencies that international students find less challenging to develop compared with local students?
   1. What are they?
   2. What factors contribute to these differences?
2. Do you feel there are COMPASS® competencies that international students find more challenging to develop compared with local students?
   1. What are they?
   2. What factors contribute to these differences?
3. Do you feel that students’ English language skills have influenced their competency development on placement?
   - 1. Why?
     2. What has been your experience of this?
4. Do you feel that cultural factors have influenced competency development on placement?
   - 1. Why?
     2. What has been your experience of this?

Primary:

1. What have been your experiences of assessing International students on placement compared with local students?
   1. Do you feel being a local or international student influences assessment on placement?
      1. How?
   2. Do you do anything differently when assessing international students compared with local students?

Secondary:

1. How do you feel about assessing international students compared with local students?
2. What are your experiences of assessing international students using COMPASS®?
3. Do you feel that there are areas of competency not assessed by COMPASS® for international students?

Primary:

1. Could you give examples of strategies that you’ve used that you feel have helped international students on placement?
   1. How have they helped?
   2. What sort of support have you found helpful for you when having international students on placement?

Primary:

1. Is there anything else about having international students on placement that you would like to share?
